# Kinetic Study and Catalytic Activity of Cr^3+^ Catalyst Supported on Calcium Silicate Hydrates for VOC Oxidation

**DOI:** 10.3390/ma17143489

**Published:** 2024-07-14

**Authors:** Ramune Sidaraite, Kestutis Baltakys, Andrius Jaskunas, Nedas Naslenas, Darius Slavinskas, Edvinas Slavinskas, Tadas Dambrauskas

**Affiliations:** 1 Department of Silicate Technology, Kaunas University of Technology, Radvilenu 19, LT-50270 Kaunas, Lithuania; ramune.sidaraite@ktu.lt (R.S.); tadas.dambrauskas@ktu.lt (T.D.); 2 Department of Physical and Inorganic Chemistry, Kaunas University of Technology, Radvilenu 19, LT-50270 Kaunas, Lithuania; andrius.jaskunas@ktu.lt (A.J.); nedas.naslenas@ktu.lt (N.N.); 3 JSC “Lagūna”, Bajoriskiu 13, LT-36159 Panevezys, Lithuania; 4 JSC “Bio-Techno Line”, Juostos 5, Trakiskis, LT-38102 Panevezys, Lithuania

**Keywords:** chromium, calcium silicate hydrates, volatile organic compounds, kinetic, catalyst

## Abstract

Volatile organic compounds (VOCs) are pollutants that pose significant health and environmental risks, necessitating effective mitigation strategies. Catalytic oxidation emerges as a viable method for converting VOCs into non-toxic end products. This study focuses on synthesizing a catalyst based on calcium silicate hydrates with chromium ions in the CaO-SiO_2_-Cr(NO_3_)_3_-H_2_O system under hydrothermal conditions and evaluating its thermal stability and catalytic performance. A catalyst with varying concentrations of chromium ions (10, 25, 50, 100 mg/g Cr^3+^) was synthesized in unstirred suspensions under saturated steam pressure at a temperature of 220 °C. Isothermal curing durations were 8 h, 16 h, and 48 h. Results of X-ray diffraction and atomic absorption spectroscopy showed that hydrothermal synthesis is effective for incorporating up to 100 mg/g Cr^3+^ into calcium silicate hydrates. The catalyst with Cr^3+^ ions (50 mg/g) remained stable up to 550 °C, beyond which chromatite was formed. Catalytic oxidation experiments with propanol and propyl acetate revealed that the Cr^3+^ catalyst supported on calcium silicate hydrates enhances oxygen exchange during the heterogeneous oxidation process. Kinetic calculations indicated that the synthesized catalyst is active, with an activation energy lower than 65 kJ/mol. This study highlights the potential of Cr^3+^-intercalated calcium silicate hydrates as efficient catalysts for VOC oxidation.

## 1. Introduction

Environmental pollution causes various negative effects not only on living organisms but also on plant life [1]. Pollutants can persist in the environment for extended periods, causing more significant and widespread environmental damage [2]. One of the main air pollutants is volatile organic compounds (VOCs) [3]. VOCs are various organic chemicals that have a high vapor pressure at room temperature [4]. They typically contain elements such as hydrogen, oxygen, chlorine, bromine, sulfur, fluorine, or nitrogen, and are mostly emitted during the production of various organic compounds (paints, glues, solvents, additives, etc.) and the burning of fuels such as gasoline, wood, coal, or natural gas [5]. Due to their negative effect on the environment and human health, it is important to find ways to mitigate their concentration. Various research efforts have been directed towards technologies to remove VOCs from the environment and decrease their emissions from industry.

VOCs removal methods can be classified into two main categories: recovery methods and destructive methods. Recovery methods are technologies that recover VOCs through physical separation [6], such as adsorption [7], absorption [8], condensation [9], and membrane separation [10]. These technologies are effective for recovering valuable VOCs but are expensive, energy-inefficient, complex, and often create secondary waste [6]. Destructive methods convert VOCs into carbon dioxide and water, i.e., harmless end products, via various chemical and biological processes. These technologies include thermal and catalytic incineration [11], photolysis [12], catalytic combustion [13], electrochemical oxidation [14], microwave-assisted catalysis [15], photocatalytic decomposition [16], biodegradation [17], and catalytic oxidation [18]. Each method has advantages and disadvantages; however, one of the most effective and economically feasible methods is catalytic oxidation. Using this method VOCs are oxidized using suitable catalysts at much lower temperatures (250–500 °C) compared to thermal oxidation processes [19]. Catalytic oxidation also works at low concentrations and with large amounts of VOCs. Usually, catalysts used for VOC oxidation are noble-metal-based, such as platinum, gold, and palladium [20]. Unfortunately, these metals are very expensive and have limited availability, creating a demand for catalysts based on transition metals.

Chromium is a heavy metal known to be a transitional element with many industrial uses [21]. Chromium-based catalysts are well known in organic compound production. They are essential for ethylene polymerization and are widely applied in the industrial production of polyethylene and 1-hexene [22]. Chromium-based catalysts show high structural diversity while also being selective and active [23]. Typically, these catalysts consist of chromium supported on oxide with a high surface area and porosity, most often silica [22,24]. Calcium silicates and calcium silicate hydrates can be considered as innovative and environmentally friendly catalyst supports because, after usage, they can be used as additives for ordinary Portland cement, thereby avoiding secondary pollution by landfilling spent catalysts [25,26]. Calcium silicate hydrates (CSH) are silicic acid salts, whose basicity depends on the calcium oxide (CaO) and silicon dioxide (SiO_2_) ratio, marked as C/S. This system has an exceptional level of structural complexity. It is known that there are more than 40 crystalline calcium silicate hydrate phases with C/S ratios varying from 0.44 to 3 [27,28]. Due to the varying basicity of CSH, it is possible to create different chromium-based catalysts using a diverse range of C/S ratios. However, research on chromium catalysts supported by calcium silicate hydrates is scarce [29,30]. Niuniavaite et al. [29] investigated the catalytic activity of semi-crystalline calcium silicate hydrate (CaO/SiO_2_ = 1.5) with intercalated chromium ions for propanol oxidation, determining that a 95% conversion degree of propanol into carbon dioxide was reached at around 240 °C.

Even though there are studies on chromium-based catalysts with CSH supports, there appears to be a lack of kinetic research on the effectiveness of such products. Highlighting the novelty, this research addresses the gap by investigating the kinetic parameters of VOC oxidation using a chromium catalyst supported on CSH. We used a lower molar ratio of CaO/SiO_2_ (1.0) to synthesize more stable calcium silicate hydrates, aiming to enhance the stability and efficiency of the catalyst. Unlike previous work, this study includes detailed kinetic calculations, such as determining the Arrhenius constant and activation energy, which are crucial for understanding the catalytic mechanisms and for the future evaluation and optimization of the catalyst. While the previous study focused on propanol [29], this study extends the investigation to other VOCs, providing a broader scope of application for the catalyst. Understanding these kinetic parameters is essential for optimizing catalytic processes, enabling better modeling of VOC behavior, optimizing industrial processes, and contributing to the development of safer and more efficient catalytic systems for VOC mitigation [19,31].

Therefore, the aim of this work was to synthesize a catalyst based on calcium silicate hydrates and chromium ions in the CaO-SiO_2_-Cr(NO_3_)_3_-H_2_O system under hydrothermal conditions and determine its thermal stability and catalytic activity.

## 2. Materials and Methods

### 2.1. Materials and Synthesis

In this work, the following reagents were used:

- Calcium carbonate (“ACS reagent”, CAS 471-34-1). Prior to synthesis, calcium carbonate was ground for 5 min in a vibrating cup mill (Pulverisette 9, Fritsch, Pittsboro, NC, USA) (speed: 700 rpm) and calcined at 900 °C for 2 h. The quantity of free CaO was 99.88 mass %.
- Amorphous silicon dioxide (“Eurochemicals”, Vilnius, Lithuania) was ground for 2.5 min in a vibrating cup mill (Pulverisette 9, Fritsch, Pittsboro, NC, USA) (speed: 900 rpm). Surface area Sa = 1291 m^2^/kg, loss of ignition = 15.26%, concentration of SiO_2_ = 84.24%.
- Chromium nitrate solution prepared with concentrations of 2.5, 5.0, and 10.0 g Cr^3+^/dm^3^ by dissolving Cr(NO_3_)_3_·9H_2_O granules (“Penta”, Katovice, Czech Republic, purity = 99%) in distilled water.

Hydrothermal synthesis: For the synthesis, the mixture of amorphous silicon dioxide and calcium oxide was used with the molar ratio of CaO/SiO_2_ equal to 1.0. The dry primary mixture was mixed with a chromium nitrates solution to reach a water-to-solid ratio of 10.0:1.0. The metal ion content for 1 g of the solid mixture was 10, 25, 50, or 100 mg. The hydrothermal synthesis was carried out in unstirred suspensions in 25 mL polytetrafluoroethylene cells, placed in a stainless-steel autoclave (Moline, IL, USA), under saturated steam pressure at a temperature of 220 °C. Isothermal curing durations were 8 h, 16 h, and 48 h. and additional argon gas pressure of 4 bar was used. The temperature was reached within 2 h. After hydrothermal treatments, the autoclave was quenched to room temperature. The suspensions were filtered, and the products were rinsed with acetone to prevent carbonization, dried at 80 °C ± 5 for 24 h, and sieved (<80 μm).

### 2.2. Methods

A Nabertherm LH 15/13 (Nabertherm GmbH, Bremen, Germany) high-temperature furnace was used for the calcination of the synthesis products in a temperature range of 250–1000 °C, at a heating rate of 500 °C per hour, with a 1 h duration at the selected temperature.

Phase composition of synthesis products was determined using X-ray diffraction (XRD) powder analysis on a D8 Advance diffractometer (Bruker AXS, Karlsruhe, Germany) operating at a tube voltage of 40 kV and tube current of 40 mA. The X-ray beam was filtered with Ni 0.02 mm filter to select the CuKα wavelength. Diffraction patterns were recorded in a Bragg–Brentano geometry using a fast-counting detector, Bruker LynxEye (Bruker AXS, Karlsruhe, Germany), based on silicon strip technology. Specimens were scanned over a range of 3–70° (2θ) at a scanning speed of 6°/min using a coupled two theta/theta scan type.

XRD spectra were used to calculate the degree of crystallinity. For the calculations, Topas 4.1 software (Bruker AXS, Karlsruhe, Germany) and the following equations were used:

(1) $$Amorphous,\%=\frac{Global\;area-Reduced\;area}{Global\;area}\cdot 100\%$$

(2) Crystallinity,%=100%−Amorphous,%

The measurements of thermal stability conducted using a Linseis PT1000 instrument (Linseis Massgeraete GmbH, Selb, Germany) were under the following conditions: heating rate of 15 °C/min, temperature range 30–1000 °C, nitrogen atmosphere, ceramic sample handlers, platinum crucibles, and sample mass of approximately 13 mg.

The concentration of Cr^3+^ ions was determined using a Perkin-Elmer Analyst 4000 atomic absorption spectrometer (Perkin Elmer, Waltham, MA, USA) with parameters as follows: wavelength—357.87 nm, hollow cathode lamp current (I)—30 mA, flame type—C_2_H_2_–air, oxidant air flow—10 L/min, and acetylene flow = 2.5 L/min. All tests were repeated three times.

Propanol (purity > 98%) and propyl acetate (purity > 98%) were used as the comparative volatile organic compounds (VOCs) for the catalytic oxidation experiments. The experiments were conducted using 0.2 g of catalyst placed inside a fixed-bed quartz reactor with a coil preheater operating in steady state conditions. This reactor was housed within a Nabertherm tube furnace LH 15/13 (Nabertherm GmbH, Bremen, Germany) to ensure a stable temperature, which was accurately monitored by a K-type thermocouple positioned inside the reactor. The reactor’s inlet and outlet were equipped with specialized points for collecting gas flow samples and measuring CO and CO_2_ concentrations, which were directly connected to a TESTO 445 unit (Testo, Titisee-Neustadt, Germany). The catalytic oxidation process was carried out with various airflow rates ranging between 200 and 370 mL/min, saturated with VOC concentrations ranging from 800 to 1000 ppm. The concentration of VOCs in the gas stream was analyzed using a Perkin Elmer Clarus 500 GC/MS system (Perkin Elmer, Waltham, MA, USA), fitted with a COL-ELITE 5MS (Perkin Elmer, Waltham, MA, USA) capillary column that was 30 m in length and 0.25 mm in internal diameter. Standards of VOCs were prepared by evaporating a measured volume of liquids in a measured volume of air.

## 3. Results and Discussion

### 3.1. Hydrothermal Synthesis of Calcium Silicate Hydrates with Intercalated Cr^3+^ Ions

**Figure 1:**
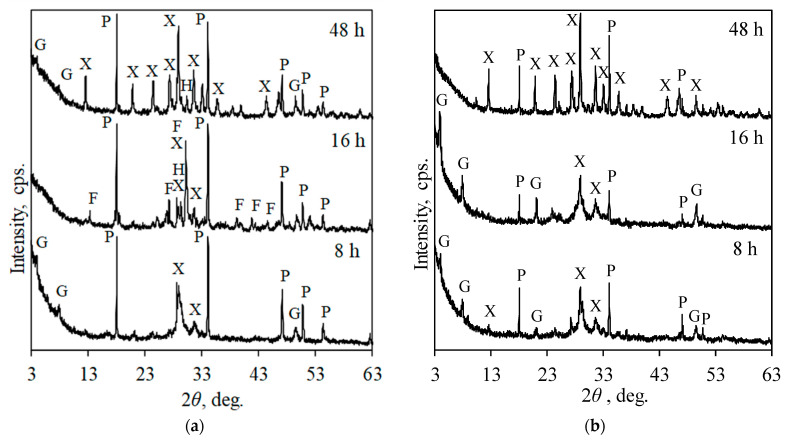
XRD patterns of synthesis products formed in pure (**a**) and with 25 mg/g of Cr^3+^ ions (**b**) systems under hydrothermal synthesis conditions. Indexes: G—gyrolite; P—portlandite; X—xonotlite; F—foshagite; H—hillebrandite.

According to the scientific literature [32], the formation of calcium silicate hydrates depends on the synthesis conditions and the nature of the raw materials. Thus, to determine the influence of Cr^3+^ ions on the formation of calcium silicate hydrates, firstly, the formation of calcium silicate hydrates in a pure calcium oxide, silicon dioxide, and water system was investigated. The phase composition of the synthesis products was determined by XRD analysis, with results presented in Figure 1.

It was determined that after 8 h of synthesis at 220 °C, a mixture of lower basicity calcium silicate hydrates, xonotlite (Ca_6_Si_6_O_17_(OH)_2_), tobermorite (Ca_5_Si_6_O_17_(H_2_O)_5_), and gyrolite (Ca_4_Si_6_O_15_(OH)_2_·3H_2_O), was formed (Figure 1a). It is worth mentioning that semicrystalline calcium silicate hydrate C-S-H(I) can also form in the synthesis products; however, its diffraction peaks overlap with other CSH phases. Additionally, under these conditions, intensive diffraction peaks characteristic of unreacted portlandite were identified.

Prolonging the synthesis duration to 16 h led to the formation of the higher basicity calcium silicate hydrates, hillebrandite (Ca_2_SiO_3_(OH)_2_) and foshagite (Ca_4_Si_3_O_9_(OH)_2_), in the synthesis product (Figure 1a). Small diffraction peaks characteristic of xonotlite were still identified. It is worth noting that the molar ratio of CaO/SiO_2_ in hillebrandite (Ca/SiO_2_ = 2.0) and foshagite (Ca/SiO_2_ = 1.33) is higher compared to the initial mixture (Ca/SiO_2_ = 1.0). This is likely due to lower reactivity and solubility of amorphous silicon dioxide compared to calcium oxide, resulting in an excess of calcium oxide in the liquid medium, which led to the formation of higher basicity silicates. Similar results have been presented in the literature [27]. As expected, prolonging the synthesis duration to 48 h negatively affected the stability of hillebrandite and foshagite (Figure 1a). Foshagite fully and hillebrandite partially recrystallized into xonotlite, whose molar ratio corresponds to the initial mixture. Additionally, small-intensity diffraction peaks characteristic of gyrolite were also identified.

Cr^3+^ ions strongly affected the phase composition of synthesis products (Figure 1). XRD results showed that, after 8 h of synthesis, in the system with 25 mg/g of Cr^3+^ ions, a mixture of calcium silicate hydrates, xonotlite, tobermorite, and gyrolite, was formed (Figure 1b). Although the phase composition of the product was the same as in the pure system, the intensity of diffraction peaks was significantly higher in the system with chromium ions. On the other hand, the diffraction peaks characteristic of portlandite were less intensive. The intensity of diffraction peaks characteristic of all formed calcium silicate hydrates increased while those of portlandite decreased with prolonged synthesis duration at 16 h. Finally, after 48 h of synthesis, xonotlite was the only calcium silicate hydrate formed in the products (Figure 1b). However, quite intensive diffraction peaks characteristic of unreacted portlandite were still identified in the XRD pattern.

The results of atomic absorption spectroscopy showed that the concentration of Cr^3+^ ions in the liquid medium obtained after synthesis slightly depends on the duration of synthesis. It was measured that, after 8 h and 16 h of synthesis, the concentration of Cr^3+^ ions in the liquid medium was 1.67 mg/L and 3.7 mg/L, respectively. Since the initial concentration of metal ions in the liquid medium was 2500 mg/L, it can be stated that only an insignificant part of these ions was not intercalated into the structure of calcium silicate hydrates. Due to formation of a highly crystalline calcium silicate hydrate—xonotlite—the concentration of Cr^3+^ ions in the liquid medium increased to 42.7 mg/L. However, the amount of these ions in the liquid medium still did not exceed 2% of the initial amount. It can thus be stated that during hydrothermal synthesis, more than 98% of Cr^3+^ ions were incorporated into the structure of the synthesis product.

Further analysis of the liquid medium showed that the amount of calcium ions released from solid compounds increased from 9.7 mg/g to 16.2 mg/g with an increase in synthesis duration from 8 h to 48 h. It was calculated that the moles of intercalated chromium ions (25 mg = 0.48 mmol) correspond to the moles of released calcium ions (0.24–0.41 mmol). Probably, during synthesis, the chromium ions replaced calcium ions in the structure of calcium silicate hydrates. Similar findings have been presented in the literature [33].

**Figure 2:**
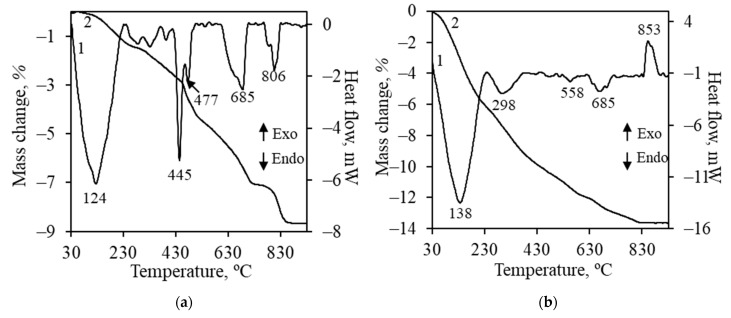
STA curves (1—DSC; 2—TGA) of synthesis products formed in system with 50 mg/g of Cr^3+^ ions (48 h) (**a**) and 100 mg/g of Cr^3+^ ions (16 h) (**b**) under hydrothermal synthesis conditions.

The simultaneous thermal analysis results of synthetic xonotlite with intercalated chromium ions are presented in Figure 2a. The first intensive endothermic effect at 124 °C can be assigned to the removal of adsorbed water. The second intensive doublet at 445 °C and 477 °C can be attributed to the dehydration of unreacted portlandite. The third effect (685 °C), during which the sample lost 1.6% of its mass, can be assigned to the decomposition of calcium carbonate or the formation of chromium-containing compounds. Theoretically, based on mass loss, the possible amount of calcium carbonate in the system is lower than 3.6%, thus. due to the low amount and overlapping of peaks, diffraction peaks of this compound were not identified in the XRD pattern (Figure 1b). Finally, the effect at 806 °C can be assigned to solid phase sintering (Figure 2a) [34]. It is worth noting that no exothermic effect was observed in the temperature range of 800–900 °C, indicating that semicrystalline calcium silicate hydrates such as C-S-H(I) and C-S-H(II) were not formed during hydrothermal synthesis.

**Figure 3:**
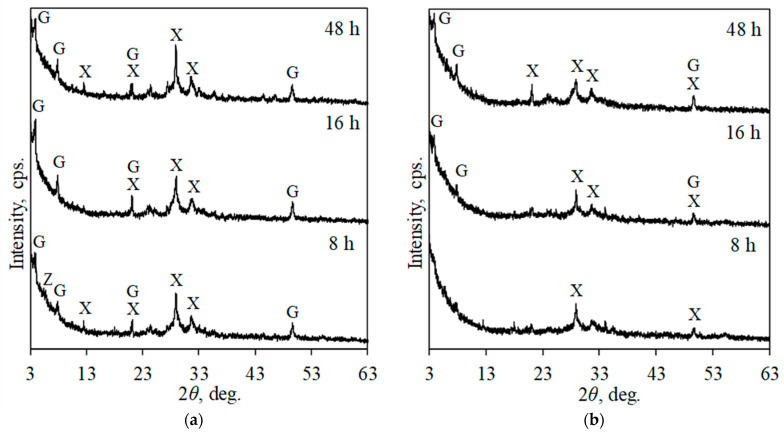
XRD patterns of synthesis products formed in the system with 50 mg/g of Cr^3+^ ions (**a**) and 100 mg/g of Cr^3+^ ions (**b**) under hydrothermal synthesis conditions. Indexes: G—gyrolite; X—xonotlite; Z—Z-phase.

Increasing the chromium ion concentration in the system to 50 mg/g positively affected the reactivity of portlandite because it was fully reacted at the beginning of synthesis (8 h) (Figure 3a). It was determined that after 8 h of synthesis, a mixture of xonotlite and lower basicity compounds (gyrolite and Z-phase) was formed. By prolonging synthesis duration to 16–48 h, Z-phase became metastable and recrystallized to xonotlite and gyrolite. It is worth mentioning that the diffraction peaks characteristic of these compounds were quite low in intensity compared to previously discussed systems. The analysis of the liquid medium showed that, despite the duration of synthesis, all Cr^3+^ ions (50 mg/g) were intercalated into the structure of the synthesis products because their concentration in the liquid medium did not exceed the detection limit of AAS. Meanwhile, the concentration of calcium ions was 3680 mg/L, corresponding to 36.8 mg of Ca^2+^ per gram of the solid mixture. It was calculated that 0.96 mmol of chromium was combined per gram of solid material and 0.92 mmol of calcium was released.

The obtained results are in good agreement with the STA data (Figure 2b). The first effect can be attributed to the removal of adsorbed water and partial dehydration of gyrolite. The second effect (298 °C) is not typically associated with either gyrolite or xonotlite. It is probably related to the removal of intercalated nitrate anions from the structure of gyrolite [35] or dehydration of amorphous compounds. Small endothermic effects at 558 °C and 685 °C are related to the formation of compounds containing chromium ions and the decomposition of calcium carbonate, respectively. The exothermic effect at 853 °C is typical of the recrystallization of lower basicity calcium silicate hydrates to wollastonite.

Further increasing the Cr^3+^ ion concentration to 100 mg/g negatively affected the formation of CSH because, after 8 h and 16 h of synthesis, only traces of xonotlite and gyrolite were obtained (Figure 3b). After 48 h of synthesis, the intensity of diffraction peaks characteristic of gyrolite and xonotlite increased; however, they remained of low intensity. The analysis of the liquid medium showed that the concentration of Cr^3+^ ions in the liquid medium was lower than 10 mg/L under all experimental durations. Thus, more than 99.9% (99.9 mg/g) of chromium ions were combined by the synthesis products. As in the previous case, the moles of intercalated chromium ions (1.92 mmol) correspond to the moles of calcium ions released into the liquid medium (1.93 mmol).

Summarizing the obtained data, it is possible to state that ion exchange reactions between calcium and chromium proceeded during the hydrothermal synthesis of calcium silicate hydrate. As a result, the molar ratio of CaO/SiO_2_ in the final product decreases, leading to the formation of lower basicity CSH. This is in good agreement with data in the literature, which observed that CSH with a molar ratio of CaO/SiO_2_ lower than 1.0 is formed in the mixtures with CaO/SiO_2_ = 1.5 [29].

### 3.2. Thermal Stability of Calcium Silicate Hydrates with Intercalated 50 mg/g of Cr^3+^ Ions

The thermal stability of catalysts is a crucial parameter that can determine their potential application. For this reason, the sample obtained after 16 h of synthesis in the mixture with 50 mg/g of Cr^3+^ ions was calcined in the temperature range of 250–1000 °C. This sample was chosen because portlandite was fully reacted and two stable calcium silicate hydrates (gyrolite and xonotlite) were formed.

**Figure 4:**
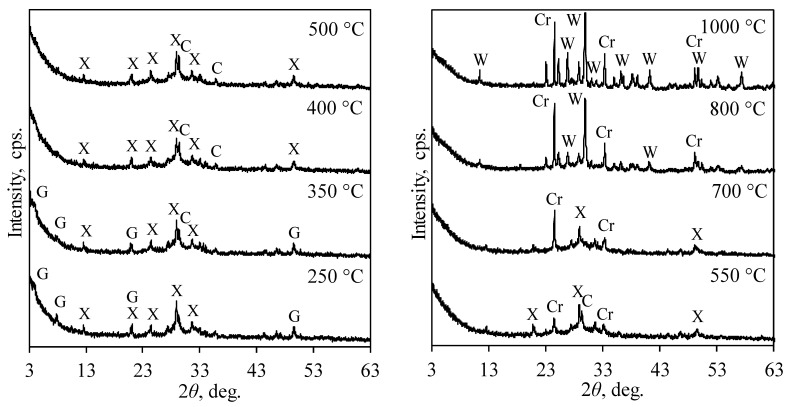
XRD patterns of calcined products (Cr^3+^—50 mg/g, 220 °C, 16 h) at different temperatures. Indexes: G—gyrolite; X—xonotlite; Cr—chromatitte; W—wollastonite; C–calcium carbonate.

It was determined that after calcination at 250 °C, the intensity of diffraction peaks characteristic of gyrolite decreased due to the removal of interlayer water (Figure 4) [36]. A further increase in temperature to 400 °C led to the full dehydration of gyrolite. It is worth noting that during dehydration of gyrolite, truscottite, which has diffraction peaks close to those of gyrolite, is formed [36]. However, in XRD spectra of products calcined at 250–350 °C, truscottite was not identified due to the low intensity of diffraction peaks and insignificant shifts in their position. Probably, this is the result of the intercalated chromium ions in the structure of gyrolite.

After calcination at 550 °C, chromium ions reacted with calcium silicate hydrates and formed calcium chromatite (CaCrO_4_) (Figure 4). Similar results have been obtained by other authors in similar systems [37]. It is worth mentioning that the formation of chromatite leads to the decrease in the catalytic activity of synthesis products for VOCs [29]. It was determined that a further increase in calcination temperature to 700 °C led to further dehydration of xonotlite (only the main peak was identified) and an increase in intensity of diffraction peaks characteristic of chromatite. Meanwhile, after calcination at 800 °C, xonotlite fully dehydrated and wollastonite (CaSiO_3_) was formed. Finally, after calcination at 1000 °C, intense diffraction peaks characteristic only of wollastonite and chromatite were identified in the XRD pattern (Figure 4).

**Figure 5:**
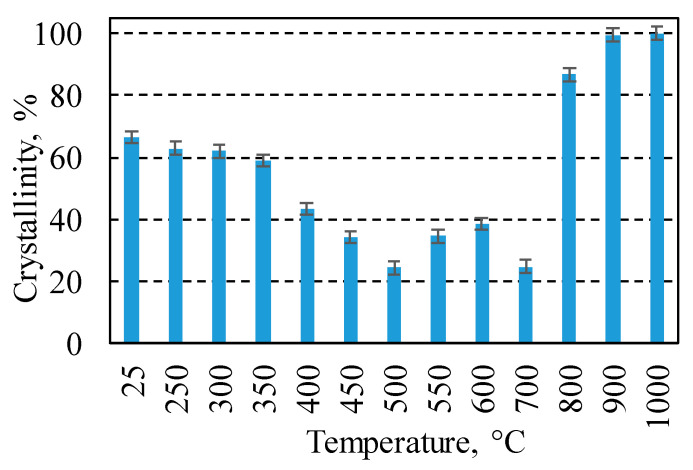
Crystallinity of calcined products (Cr^3+^—50 mg/g, 220 °C, 16 h) at different temperatures.

The temperature of calcination influences the crystallinity of materials, which can determine the activity of materials. For the calculations of crystallinity, the global area and reduced area of XRD patterns was calculated using Topas 4.1 software. Using these values, crystallinity was calculated by Equations (1) and (2), and the obtained data are presented in Figure 5. It was calculated that the crystallinity of the sample obtained after synthesis was 66.5%. This value slightly decreased to 59% with an increase in calcination temperatures up to 350 °C. The decrease in crystallinity is related to the removal of adsorbed water and partial dehydration of gyrolite (Figure 2b and Figure 4). Due to the full dehydration of gyrolite and partial dehydration of xonotlite, a sharp decrease in crystallinity (to 24.4%) was observed at temperatures from 400 °C to 500 °C (Figure 5). The formation of crystalline chromatite led to the increase in crystallinity to 38.6% at 600 °C; however, this value decreased to 25% at 700 °C. The second decrease in crystallinity can be explained by the dehydration of xonotlite. The formation of wollastonite at temperatures above 800 °C led to an increase in crystallinity, and after calcination at 1000 °C, only crystalline phases were present in the sample.

### 3.3. Catalytic Activity of Calcium Silicate Hydrate with Intercalated Cr^3+^ Ion

The catalytic activity of the synthesized (220 °C, 16 h) and additionally calcined (350 °C) sample was evaluated through the complete oxidation of propanol and propyl acetate in an air stream. A calcination temperature of 350 °C was chosen to obtain a stable structure of the catalyst in the investigated temperature interval. Meanwhile, propanol and propyl acetate were chosen for comparison to identify the more suitable candidate for subsequent kinetic experiments. Since the catalytic activity is highly dependent on the type of VOC, selecting a contaminant that can be completely oxidized within the operating temperature range is advantageous. The primary product of complete oxidation is carbon dioxide, making the key performance parameter the reduction in VOC concentration relative to CO_2_ formation. Due to slight variations in the initial concentration of contaminants, all measured concentration values were normalized and are reported per gram of catalyst per gram of propanol or propyl acetate in the incoming stream. The reduction in VOC concentration is presented as a conversion, in percentage units, while the selectivity of the catalysts was assessed based on the amounts of intermediates detected in the outgoing flow. The experiments were conducted within a temperature range of 150 to 300 °C, with the temperature increasing by approximately 25 °C every hour.

**Figure 6:**
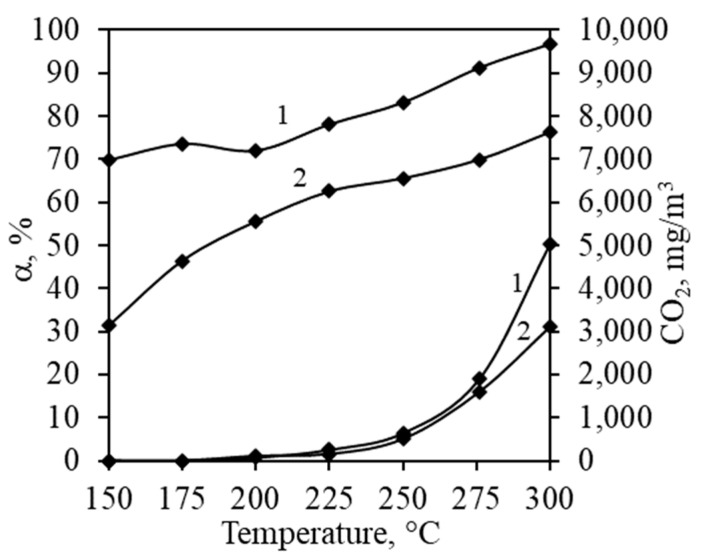
Conversion and produced amounts of CO_2_ during complete oxidation of propyl acetate (1) and propanol (2) at various temperatures.

At an initial temperature of 150 °C, both samples exhibited apparent performance in reducing the concentration of VOCs (Figure 6). However, this reduction can be attributed to adsorption rather than catalytic oxidation, as indicated by the absence of CO_2_ in the outgoing stream. The catalyst showed greater adsorptive affinity towards propyl acetate, with a reduction of 69% in its concentration compared to 31% for propanol. This could be explained by the higher boiling point and molar mass of propyl acetate. Adsorption influenced the overall process even at temperatures up to 250 °C. As the temperature in the catalyst bed increased, there were sharp rises in CO_2_ concentrations and sudden temperature spikes due to the exothermic nature of the oxidation reaction. CO_2_ production began to increase at 200 °C for both volatiles, indicating similar light-off temperatures. This was supported by the appearance of CO in the outgoing stream, suggesting that incomplete catalytic oxidation reactions were occurring alongside the formation of CO_2_. The catalyst demonstrated higher catalytic activity towards propyl acetate, evidenced by a sharper increase of CO_2_ concentration. The convergence of the conversion and CO_2_ accumulation curves suggests a transition from adsorption to catalytic oxidation with rising temperature. The catalyst achieved 97% conversion at around 300 °C for propyl acetate, while for propanol, it reached only 76%. This indicated that propyl acetate would be the better option for kinetic experiments because it achieved higher conversion and, with a slight increase of temperature, the complete oxidation of VOCs will occur.

**Figure 7:**
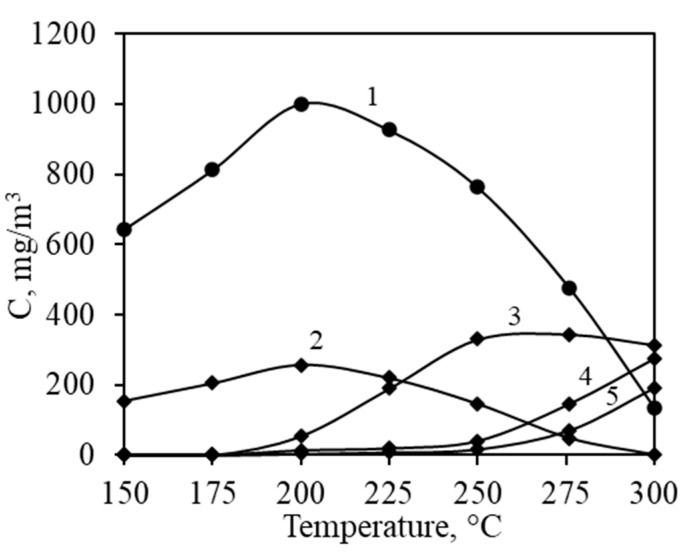
Intermediates produced during complete oxidation of propyl acetate and propanol: 1—propanol; 2—acetic acid; 3—isopropanol; 4—CO (propanol); 5—CO (propyl acetate).

Monitoring the outgoing gas stream with a CO probe and GC/MS revealed the presence of incomplete catalytic oxidation products, specifically intermediates (Figure 7). Carbon monoxide (CO) typically forms at the onset of catalytic oxidation and is rapidly oxidized to CO_2_ as the temperature rises. Thus, CO formation can serve as an indicator of catalytic activity. The synthesized sample began producing CO at the same temperatures for both propanol and propyl acetate, peaking at 273 and 190 mg/m^3^ at 300 °C, respectively. By comparing CO formation curves, it is clear that the oxidation of propyl acetate produced less CO, reflecting its higher apparent selectivity.

However, GC/MS analysis indicated more intermediates were produced during the complete oxidation of propyl acetate, namely propanol and acetic acid (Figure 7). Both intermediates formed because of a hydration reaction on the surface of the catalyst, and their formation was detected at the initial temperature of 150 °C. This indicates that the hydration reaction does not require high activation energy and is probably favored by adsorption. Both compounds reached their peak concentrations at 200 °C and were completely oxidized together with propyl acetate when the temperature was increased. It is noticeable that acetic acid was apparently oxidized much more easily on the surface of the catalyst, as its detected concentrations were three times lower. This suggests that acetic acid has a higher oxidation rate compared to propanol, contributing to the overall efficiency of propyl acetate oxidation.

The catalytic oxidation of propanol produces a peculiar intermediate—isopropanol. Isopropanol formation occurred at the same point where catalytic oxidation overtook adsorption, with a peak concentration at 275 °C (Figure 7). Isopropanol is more challenging to oxidize than propanol, thus its concentration decreased only slowly with increasing temperature in the catalyst bed, not disappearing from the stream even at 300 °C. Isopropanol forms through the interaction between propanol and the catalyst surface, specifically via the dehydration of propanol. The results of the catalytic oxidation of both compounds indicate that water vapor played a crucial role in the reactions occurring on the surface of the catalyst. Structural water acted in cycles in both hydration and dehydration reactions. Since higher conversion was achieved for propyl acetate oxidation, this volatile was used for kinetic experiments.

### 3.4. Kinetics of Propyl Acetate Complete Oxidation Reaction on the Surface of Calcium Silicate Hydrate with Intercalated Cr^3+^ Ions

**Figure 8:**
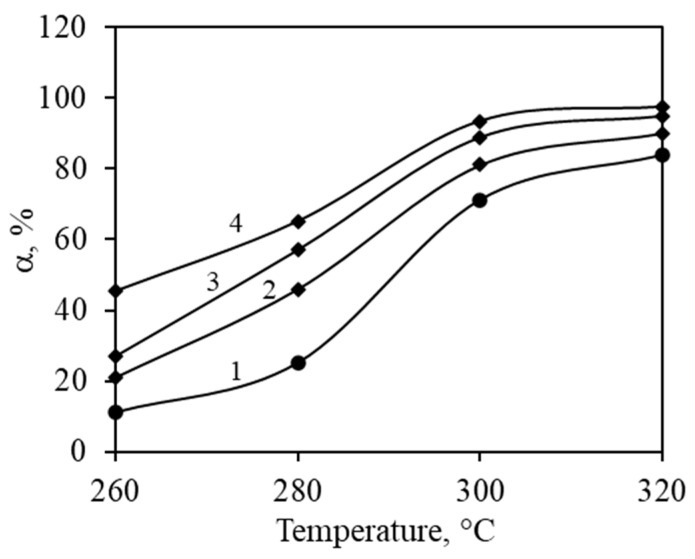
Propyl acetate conversion dependency on temperatures at various contact times: 1—3.79 s; 2—5.17 s; 3—6.23 s; 4—7.44 s.

The kinetic parameters of propyl acetate complete catalytic oxidation were determined with a constant concentration stream (1000 ppm) flowing through a catalyst bed at varying flow rates of 200–370 mL/min. Flows for catalytic oxidation were prepared by mixing vapors of VOCs into an air stream. By adjusting the flow rates of these streams, desired concentrations of VOCs were achieved, which were analyzed by GC/MS. These flow rates were used to calculate the contact duration of propyl acetate, which ranged from 3.79 to 7.44 s (Figure 8). By varying the flow rates and hence the contact durations, the relationship between these variables and the catalytic oxidation efficiency was observed. As expected, longer contact times allow for more complete oxidation of propyl acetate due to prolonged exposure to the catalyst surface. Additionally, higher temperatures facilitate more efficient catalytic reactions, contributing to the overall increase in conversion rates.

These experiments were used to determine reaction rate constants and to assess the activation energy of complete oxidation. According to the law of mass action, the rate of oxidation of propyl acetate is directly proportional to the concentrations of the volatile compound and oxygen:

(3) $$r=kC_{PA}^{n}C_{O_{2}}^{m}$$

where *r*—reaction rate, *C_PA_*—concentration of propyl acetate, *C_O_*_2_—concentration of oxygen, and *n* and *m*—partial orders of reaction.

Since oxygen is comparatively in excess, its concentration change is negligible, so it can be assumed that the reaction rate does not depend on it. The reaction can be calculated as a pseudo first order reaction.

(4) $$r=-\frac{dC_{PA}}{dt}=kC_{PA}$$

When integrated, the equation becomes as follows:

(5) $$\ln\left(1-\frac{\alpha }{100}\right)=-kt$$

For a PFR (plug flow reactor), the contact time is a product of the flow rate of the reactant, the initial concentration of the reactant, and the void volume of the catalyst:

(6) $$t_{contact}=\frac{G}{C_{PA}\cdot V}$$

where *G*—flow rate of propyl acetate, *C_PA_*—concentration of propyl acetate, and *V*—void volume of catalyst.

**Figure 9:**
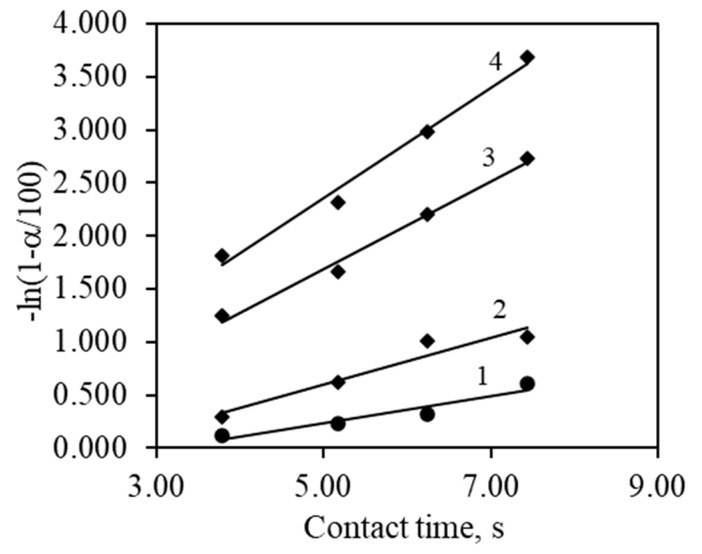
Propyl acetate oxidation kinetics in semi-logarithmic coordinates at various temperatures: 1—260 °C; 2—280 °C; 3—300 °C; 4—320 °C.

Contact times were calculated by assuming the volume of the fixed bed and a void fraction of the catalyst particle. For 30–60 µm particles, the void fraction was determined to be 0.43. Reaction rate constants can be determined by varying the contact times. The catalytic oxidation results in semi-logarithmic coordinates allow the reaction rate constants to be calculated as the slope of the received straight lines (Figure 9).

The results indicate that the reaction rate constant increases with increasing temperature, thus it can be used to determine the activation energy of the complete oxidation of propyl acetate on the surface of the catalyst. The calculated reaction rate constants are presented in Table 1.

**Figure 10:**
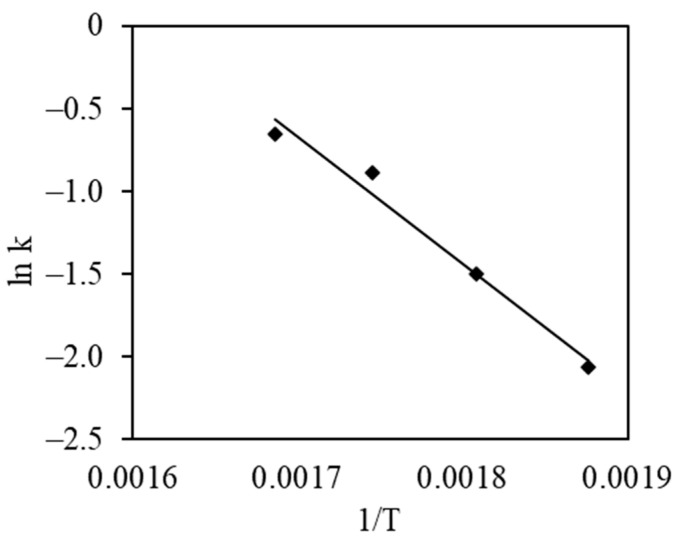
Arrhenius plot for the complete oxidation of propyl acetate on the surface of the catalyst.

Kinetic data plotted in a semi-logarithmic Arrenius plot gives a straight line whose slope can be used to calculate the activation energy of the reaction (Figure 10). The calculated activation energy of 63,847 J/mol is comparable to and falls in the middle between those of other catalysts whose activation energies usually range between 20 and 100 kJ/mol. The activation energy defines the reaction’s sensitivity to the temperature. The pre-exponent factor or Arrhenius constant defines the frequency of reactant collisions that lead to the formation of new products. The calculated Arrhenius constant was 238,948 s^−1^, which is lower when compared to other catalysts, and can be attributed to a relatively low surface area.

In comparison with the scientific literature (Table 2), the synthetic Cr^3+^ catalyst supported on calcium silicate hydrate demonstrated significant catalytic activity for VOC oxidation, specifically for propyl acetate and propanol. The synthetic catalyst achieved a 97% conversion degree for propyl acetate at 300 °C and a 76% conversion degree for propanol with an activation energy of 63.85 kJ/mol. These results are competitive with previously reported catalysts based on transition metal ions that show a conversion degree for VOCs ranging from 90% to 100% at temperatures above 240 °C (Table 2). It is worth noting that the literature is scarce regarding the reaction rate or activation energy of catalysts based on transition metal ions. The activation energy of such catalysts typically ranges around 50 kJ/mol but can exceed 250 kJ/mol.

Since the obtained data show promising results, future research will focus on further optimization of the synthesis conditions to enhance the stability and efficiency of the catalyst. Additionally, the performance of the Cr^3+^ catalyst supported on calcium silicate hydrates will be investigated with a wider range of VOCs and under different environmental conditions will be investigated for broader applications. The long-term stability and reusability of the catalyst will also be determined to allow evaluation for practical applications.

## 4. Conclusions

Cr^3+^ ions promote the reaction of portlandite and lead to the formation of lower basicity calcium silicate hydrates during hydrothermal treatment at 220 °C. In the pure system, portlandite did not fully react even after 48 h, while in the system with 50 mg/g of Cr^3+^, portlandite fully reacted after 8 h. Additionally, Cr^3+^ ions stoichiometrically replaced calcium ions in the structure of calcium silicate hydrates, resulting in the formation of lower basicity compounds. Despite the initial concentration of Cr^3+^ ions (up to 100 mg/g), the intercalation efficiency by calcium silicates hydrates was more than 98% under all experimental conditions.

The Cr^3+^ catalyst supported on calcium silicate hydrates (16 h, 220 °C, 50 mg/g) remained stable up to 350 °C during calcination in an air atmosphere. At higher temperatures, the decomposition of gyrolite (~350 °C) and xonotlite (~700 °C) and the formation of chromatite (~550 °C) and wollastonite (800 °C) proceeded. The lowest degree of catalysts crystallinity was obtained after calcination at 500 °C and 700 °C, i.e., when the sample intensively lost structural water.

The evaluation of the Cr^3+^ catalyst supported on calcium silicate hydrates revealed that the reaction with propyl acetate exhibited higher adsorptive affinity and catalytic activity compared to propanol, achieving a 97% conversion rate at 300 °C versus 76% for propanol. The reaction rate constants, calculated from semi-logarithmic plots, increased with temperature and were used to determine an activation energy of 63.847 kJ/mol.

## Figures and Tables

**Table 1:** Calculated reaction rate constants of propyl acetate oxidation at various temperatures.

Temperature, °C	k, s^−1^	ln k
260	0.127	−2.067
280	0.223	−1.501
300	0.412	−0.886
320	0.518	−0.659

**Table 2:** Activity of different catalysts for VOC oxidation.

Catalyst	Support	VOCs	Conversion Degree, %	Temperature, °C	Activation Energy, kJ/mol	Reference
Cr	-	Benzene	99	350	44.7	[38]
Cr	Betonite	Chlorobenzene	90	600	-	[39]
Cr	Semicrystalline CSH (C/S = 1.5)	Propanol	95	240	-	[29]
Pd	Betonite	Chlorobenzene	90	600	-	[39]
Co	Clay	Acetylene	100	360	55	[40]
Co	Clay	Propylene	100	460	56	[40]
Mn	-	Toluene	90	248	253.7	[41]
Mn	Clay	Propane	92	450	-	[42]
Mn	Silica	Chlorobenzene	100	500	-	[43]
Fe	Montmorillonite	Chlorobenzene	100	500	-	[44]
Cu	Semicrystalline CSH (C/S = 1.5)	Propanol	94	290	-	[45]
Cu + Co + Cr	Semicrystalline CSH (C/S = 1.5)	Propanol	97	300	-	[46]

## Data Availability

The raw data supporting the conclusions of this article will be made available by the authors on request.
